# Impact of support characteristics and preparation method on photocatalytic activity of TiO_2_/ZSM-5/silica gel composite photocatalyst

**DOI:** 10.1098/rsos.180918

**Published:** 2018-09-19

**Authors:** Emad K. Radwan, Cooper H. Langford, Gopal Achari

**Affiliations:** 1 Water Pollution Research Department, National Research Centre, 33 El Bohouth St, Dokki, Giza, Egypt, PO 12622, Egypt; 2 Department of Chemistry, University of Calgary, 2500 University Drive NW, Calgary, Alberta, Canada T2N 1N4; 3 Department of Civil Engineering, University of Calgary, 2500 University Drive NW, Calgary, Alberta, Canada T2N 1N4

**Keywords:** light-emitting diode, phenol, sulfamethoxazole, photocatalysis, adsorption, photocatalytic ozonation

## Abstract

Titanium dioxide (Degussa P25) was supported onto two different aluminosilicate zeolites (ZSM-5) and anchored on three silica gels using two separate preparation methods to study the effect of the catalyst components and the preparation method on the photoactivity of composite catalysts. The photoactivity was investigated by tracking phenol disappearance in a batch UVA light-emitting diode reactor. An easily separable photocatalyst with higher photoactivity than commercial Degussa P25 was developed using Degussa P25, ZSM-5 (SiO_2_/Al_2_O_3_ = 280) and silica gel (particle size 0.2–0.5 mm and pore size 40 Å). The optimum composition was found to be P25:ZSM-5:silica gel = 0.3 : 0.5 : 0.5 g l^−1^. SEM photographs show that the distribution of the composite catalyst components prepared without a binder was better than that prepared with a binder. The efficiency of photocatalytic ozonation of sulfamethoxazole (SMX) using the new photocatalyst was assessed and compared to that of commercially available Degussa P25. It was found that photocatalytic ozonation promoted the SMX disappearance and mineralization. PZS was superior to Degussa P25 with respect to photocatalysis and photocatalytic ozonation. The enhancement was attributed to the synergetic effect between adsorption, ozonation and/or photocatalytic oxidation.

## 1. Introduction

Heterogeneous photocatalysis continues to draw significant interest as it can degrade and mineralize certain persistent organic pollutants while enhancing biodegradability and decreasing toxicity in others [1]. By far titanium dioxide (TiO_2_) is the most widely used photocatalyst due to its high activity, mechanical and chemical stability, biocompatibility, non-toxicity, low cost and availability [1–3]. However, most commercial TiO_2_ have particle sizes in nanometre range which makes the catalyst recovery difficult and costly. Also, adsorption of pollutants on the photocatalyst surface is needed for higher effectiveness of TiO_2_ photocatalysis [4].

One approach to overcome these hindrances is to co-locate an adsorbent with TiO_2_ such that the adsorbent concentrates the pollutants adjacent to the photocatalytic sites. If binding is weak, the adsorbed pollutants diffuse between the adsorptive sites and the photocatalytic sites leading to its degradation. This mechanism is known as ‘adsorb and shuttle’ [4–7]. In addition, the composition of an adsorbent and a photocatalyst enables the degradation of low or non-polar pollutants which are poorly degraded due to their very limited adsorption on the polar TiO_2_ surface [4]. The composite photocatalyst also reduces the release of intermediates which continue to be held at the sorbent surface allowing subsequent degradation leading to eventual mineralization [4,6–8]. Research has produced a wide variety of schemes to immobilize TiO_2_ or produce active particles of sizes allowing facile separation (approx. 250 µm). The studies are too numerous to review here. However, it remains noteworthy that both higher [9–12] and lower [13–16] photoefficiency of the supported TiO_2_ catalysts compared with bare TiO_2_ has been reported.

With this background, the main goal of the present study is to develop a well-balanced, easily separable photocatalyst for application in water purification using TiO_2_-P25 and aluminosilicate zeolites (ZSM-5) as the major components that will be anchored on silica gel as an inert base. The study includes optimization of the photocatalyst composition, testing two ZSM-5 and three silica gel preparations, and comparing two different preparation methods to attain maximum photocatalytic activity. As an extension, the photocatalytic ozonation of SMX using the composite photocatalyst and commercial Degussa P25 were compared.

## 2. Material and methods

### 2.1. Materials

ACS grade phenol, SMX and methanol were purchased from Sigma-Aldrich. Commercial TiO_2_-P25 powder (denoted as P) was supplied by Degussa. Three silica gel with particle size 0.2–0.5 mm and pore size 40 Å (denoted as S), particle size 0.2–0.5 mm and pore size 60 Å (denoted as S60 Å) and particle size 40–60 µm and pore size 40 Å (denoted as S40–60 µm) were purchased from Acros Organic. Colloidal silica gel (30% (w/w) silica suspension in water) was purchased from Sigma-Aldrich. These chemicals were used as received. Two ZSM-5 zeolite samples with SiO_2_/Al_2_O_3_ = 280 (surface area 400 m^2^ g^−1^; denoted as Z) and Si/Al = 23 (surface area 425 m^2^ g^−1^; denoted as Z23) were obtained from Zeolyst International as NH_4_-ZSM-5 and calcined at 500°C for an hour to get H-ZSM-5. High performance liquid chromatography (HPLC) grade acetonitrile and water were used as mobile phases in HPLC. Deionized water (DI, resistivity 18.2 MΩ) from a Milli-Q system was used to prepare all solutions.

### 2.2. Catalysts preparation

Two different routes were pursued to immobilize the TiO_2_. The first route includes dispersing ZSM-5 and TiO_2_ separately in 20 ml methanol and sonicating for 30 min. The two suspensions were mixed followed by continued stirring for another 15 min. The silica gel powder was added during stirring, which was continued for an additional 15 min. Finally, the methanol was evaporated. The composite catalyst was dried in an oven at 100°C then calcined at 500°C for 3 h. Hereinafter, the composite catalyst prepared by this method is referred to as PZS.

The second route followed a procedure provided by Haque *et al.* [17]. In brief, a known weight of TiO_2_ and ZSM-5 were mixed together and dispersed uniformly in an appropriate amount of colloidal silica gel binder. A known weight of silica gel was magnetically stirred into the mixture for 30 min. The mixture was initially air dried followed by drying at 100°C. The granules obtained were gently crushed, screened and then washed several times with DI water. Finally, the catalyst was calcined at 600°C for 3 h. Hereinafter, the abbreviation PZSB is used to refer to the composite catalyst prepared by this method.

The distribution of TiO_2_ and zeolite on the surface of silica gel was examined by FEI XL30 scanning electron microscope (SEM) operated at 30 kV and Tecnai F20 high-resolution transmission electron microscope (TEM) operated at 200 kV.

### 2.3. Photocatalytic procedures

Phenol was selected as the model organic micro-pollutant to test the efficacy of the photocatalyst. It is not extensively adsorbed, suggesting that surface diffusion may be fast enough to deliver the substrate to the active site. The required quantity of the photocatalyst was accurately weighed and dispersed separately into a glass vial containing 20.0 ml of 50 mg l^−1^ initial concentration of phenol. The reaction vessels were placed in a circular bench scale 365 nm light-emitting diode (LED) photoreactor fabricated at the University of Calgary [18]. Light intensity in the vessel was determined to be 4.3 ± 0.2 × 10^16^ photons s^−1^ according to the ferrioxalate actinometry method [19]. After 30 min of dark stirring, the suspension was irradiated. Samples were withdrawn at pre-determined time intervals, centrifuged, filtered using a 0.2 µm syringe filter and phenol concentration was measured by HPLC.

### 2.4. Photocatalytic ozonation procedures

SMX was selected as the model pollutant in these experiments and the promising composite photocatalyst was used. The experimental set-up used in this study has been described elsewhere [20]. In brief, a flow through 365 nm LED photoreactor (average light intensity of 17.3 mW cm^−2^ as reported in [21]) and an ozone generator (SOZ-6G, A2Z Ozone Systems Inc., USA) were used for these experiments. The production of ozone used for this study was set up at 1.5 g h^−1^. The ozone input flow rate was adjusted by a flow meter to 1 l min^−1^.

A total of 1.95 g of PZS or 0.45 g of P25 was dispersed in 1.5 l of 100 mg l^−1^ initial SMX concentration in the reservoir, stirred and circulated by a peristaltic pump (LaSalle Scientific Inc, Model: 400–205) at a flow rate of 2 l min^–1^ in dark for 45 min. Then, simultaneously, the LED light was turned on and ozone was bubbled into the bottom of the reservoir by a porous diffuser. Samples were periodically taken from the reservoir to measure the residual SMX concentrations.

### 2.5. Analytical procedures

A ‘Varian pro star 210’ HPLC equipped with a PFP 100A column (Phenomenex kinetex 2.6 µ, 100 × 4.60 mm) with 20 µl injections and a 325 LC UV–vis detector was used for the analysis of compounds. Isocratic elution with a solvent mixture of 50% acetonitrile (0.1 formic acid) and 50% water (0.1 formic acid) at a flow rate of 1.25 or 1.00 ml min^−1^ was used for phenol or SMX, respectively. The detection wavelength was 254 nm or 270 nm for phenol or SMX, respectively. The dark equilibrium amount of compounds adsorbed per gram of photocatalysts (*q_e_*_(dark)_, mg g^−1^) was calculated as described in [22,23].

The photocatalytic activity was measured by the disappearance of compound. The degradation kinetics reasonably fit a first-order rate law. Consequently, relative ordering of reactivity of the synthesized photocatalysts was measured by trends in first-order rate constants (*k*, min^−1^).

#### 2.5.1. Total organic carbon analysis (TOC)

The mineralization of the compounds was studied using an Apollo 9000 combustion TOC analyzer equipped with an autosampler.

## 3. Results and discussion

### 3.1. Optimum composition of the photocatalysts

Prior to photocatalytic experiments, a series of control experiments were conducted following the procedures mentioned above. No phenol degradation was observed when samples were illuminated in the absence of photocatalysts or in the presence of silica gels and the zeolites.

Each component in the synthesized composite photocatalysts was investigated to select the optimum composition. For the sake of brevity, a detailed discussion is presented on the optimization of the PZS photocatalyst.

#### 3.1.1. TiO_2_ loading

**Figure 1.**
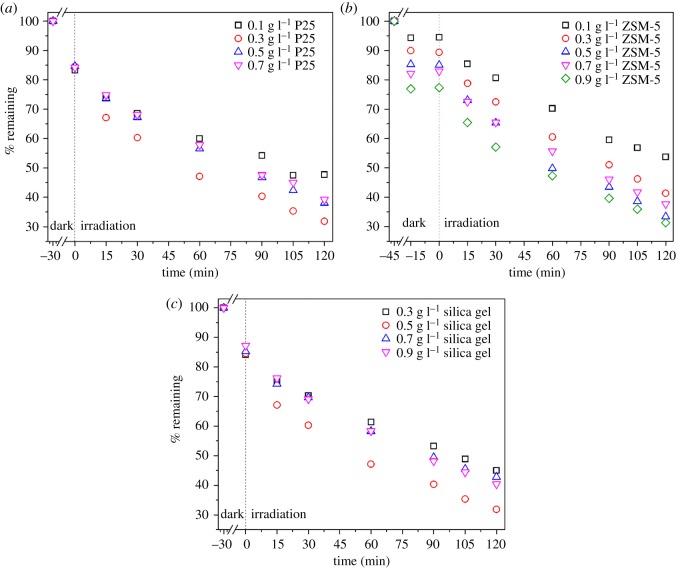
Degradation of phenol by the composite photocatalyst prepared using different loadings of (*a*) P25 (*k* = 0.0046, 0.0076, 0.0064, 0.0061 min^−1^ for 0.1, 0.3, 0.5 and 0.7 g l^−1^ P25, respectively), (*b*) ZSM-5 (*k* = 0.0047, 0.0062, 0.0074, 0.0063 and 0.0071 min^−1^ for 0.1, 0.3, 0.5, 0.7 and 0.9 g l^−1^ ZSM, respectively) and (*c*) silica gel (*k* = 0.005, 0.0076, 0.0056 and 0.0062 min^−1^ for 0.3, 0.5, 0.7 and 0.9 g l^−1^ silica gel, respectively).

Effectiveness of the PZS photocatalysts as a function of P25 content measured by degradation of phenol is presented in figure 1*a*. In these experiments, the loads of both ZSM-5 and silica gel were kept constant at 0.5 g l^–1^ while the load of P25 was changed. During the dark period, increasing the P25 loading had no effect on phenol adsorption. By contrast, during irradiation, an increase in the P25 loading to 0.3 g l^−1^ increased the phenol degradation rate due to increased active carriers (holes and hydroxyl radicals). The use of P25 over 0.3 g l^−1^ had a detrimental effect on the degradation rate, probably from light screening effects. Moreover, high catalyst loading leads to agglomeration, resulting in a reduction in surface area available for reactive sites and a consequent drop in the photocatalytic activity [1]. So, the loading of TiO_2_ was kept constant at 0.3 g l^−1^ hereafter.

#### 3.1.2. ZSM-5 loading

Figure 1*b* shows the effect of ZSM-5 loading on the photocatalytic activity of the PZS catalyst. In these experiments, the loading of P25 and silica gel was fixed at 0.3 g l^−1^ and 0.5 g l^−1^, respectively. During the dark period, significant phenol adsorption was noted for all the ZSM-5 loadings. Also, increasing the ZSM-5 loading led to an increase in the phenol adsorption due to increasing surface area [24]. Besides, leveling off in the adsorptive removal of phenol as a function of ZSM-5 loading was not observed indicating that the optimum dose of ZSM-5 was not reached. Furthermore, figure 1*b* indicates that, for each ZSM-5 loading, the amount of phenol adsorbed after 30 min was constant. After irradiation, increasing the ZSM-5 loading to 0.5 g l^−1^ led to an increase in the degradation rate of phenol. This was facilitated by the proximity of phenol molecules to TiO_2_, consequently making the phenol molecules easily reachable to the active sites on the TiO_2_ surface [7]. Increasing the ZSM-5 over 0.5 g l^−1^ has an insignificant effect on phenol degradation. It is concluded that 0.5 g l^−1^ of ZSM-5 exhibited the optimum adsorbent loading. Therefore, further experiments were performed using 0.5 g l^−1^ of ZSM-5.

#### 3.1.3. Silica gel loading

Figure 1*c* shows the effect of silica gel loading on phenol degradation. In these experiments, the loading of P25 and ZSM-5 was fixed at 0.3 g l^−1^ and 0.5 g l^−1^, respectively. Contrary to the precedent [8], increasing the silica gel content in the catalyst has no remarkable effect on phenol adsorption indicating that it does not compete as an adsorbent. This might suggest that the silica gel is completely covered with the TiO_2_ and ZSM-5 which makes it inaccessible to the phenol. Under the illumination, increasing the silica gel loading led to enhanced phenol degradation. Spreading the TiO_2_/ZSM-5 over a larger surface area, resulting in the formation of a thin layer and better utilization of TiO_2_, may have led to improvement in the photocatalytic activity. However, more addition of silica gel decreased the degradation of phenol. An optimum silica gel load of 0.5 g l^−1^ was determined.

### 3.2. Effect of supports characteristics and preparation conditions on the photocatalytic activity

**Figure 2.**
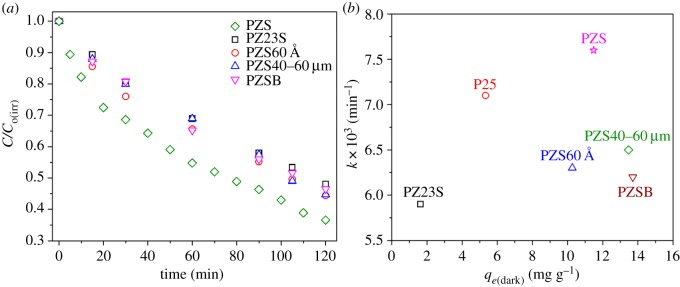
Degradation of phenol by different synthesized composite photocatalysts under UVA irradiation (*a*) comparison of photoactivity, and (*b*) rate constant (*k*) versus dark adsorption capacity, *q_e_*_(dark)_.

The composition of the photocatalysts was optimized using different zeolite and silica gel samples. The optimum composition, *k*, and *q_e_*_(dark)_ for different composite photocatalysts are shown in table 1. Figure 2*a* shows the phenol degradation by the optimum composition of the different composite photocatalysts. The time, *t* = 0 point corresponds to the normalized concentration after dark adsorption.

**Table 1.** The optimum composition, the first-order rate constants (*k*) and the dark equilibrium amount of phenol adsorbed per gram of different photocatalysts (*q_e_*_(dark)_).

photocatalyst	optimum composition P25:ZSM-5:silica gel (g l^−1^)	*q_e_*_(dark)_ (mg g^−1^)	*k* (min^−1^)
Degussa P25^a^	0.3	5.33	0.0071
PZS	0.3 : 0.5 : 0.5	11.48	0.0076
PZ23S	0.5 : 0.3 : 0.3	1.63	0.0059
PZS60 Å	0.5 : 0.3 : 0.3	10.27	0.0063
PZS40–60 µm	0.5 : 0.3 : 0.3	13.47	0.0065
PZSB	0.7 : 0.7 : 0.3 : 0.25^b^	13.71	0.0062

^a^This provides a reference point to other literature.

^b^Colloidal silica gel binder ratio.

Enhancements in the photocatalytic activity of the TiO_2_/ZSM-5 composite photocatalyst depends on the adsorption capacity, the adsorption strength [17,25,26], and the uniform distribution of the TiO_2_ and ZSM-5 over the silica gel. A photocatalyst with high adsorption capacity and weak adsorption strength will have the highest photocatalytic activity, while a photocatalyst with very strong adsorption will have the lowest photocatalytic activity. Moreover, the uniform distribution of the TiO_2_ and ZSM-5 over the silica gel ascertain better (optimum) utilization of the catalyst.

#### 3.2.1. ZSM-5 effect

SiO_2_/Al_2_O_3_ ratio is known to be one of the major factors affecting zeolite adsorptivity [17]. Therefore, ZSM-5 with different SiO_2_/Al_2_O_3_ was tested. Comparing the two composite catalysts with different zeolite samples (PZS and PZ23S) shows that the adsorption capacity and the photocatalytic activity (table 1 and figure 2) were higher with the *Z*-sample.

The high ratio of silica in the *Z*-sample increases its surface hydrophobicity which, in turn, promotes phenol adsorption [26]. Accordingly, concentrating phenol near the TiO_2_ increases the proximity and degradation. It is noteworthy that, although the PZ23S photocatalyst has higher P25 ratio, the photocatalytic activity was lower than the P25 and PZS photocatalyst. This might be due to light scattering, screening effect, agglomeration and/or formation of a thick layer of P25 over the silica gel.

#### 3.2.2. Silica gel pore size effect

Increasing the pore size of the silica gel (PZS60 Å) results in optimum composition of the composite photocatalyst with lower content of ZSM-5, consequently decreasing both the adsorption capacity and the photocatalytic activity (table 1 and figure 2).

#### 3.2.3. Silica gel particle size

The composite photocatalyst with smaller silica gel particle size (PZS40–60 µm) has a slightly higher adsorption capacity and lower photocatalytic activity (table 1 and figure 2) than the PZS photocatalyst. As mentioned earlier [17], increasing the adsorption strength decreases the photocatalytic activity. However, as the increase in adsorption capacity is insignificant, the suppressed photocatalytic activity in the PZS40–60 µm might be due to stronger adsorption characteristics induced by the silica gel.

#### 3.2.4. Preparation method effect

**Figure 3.**
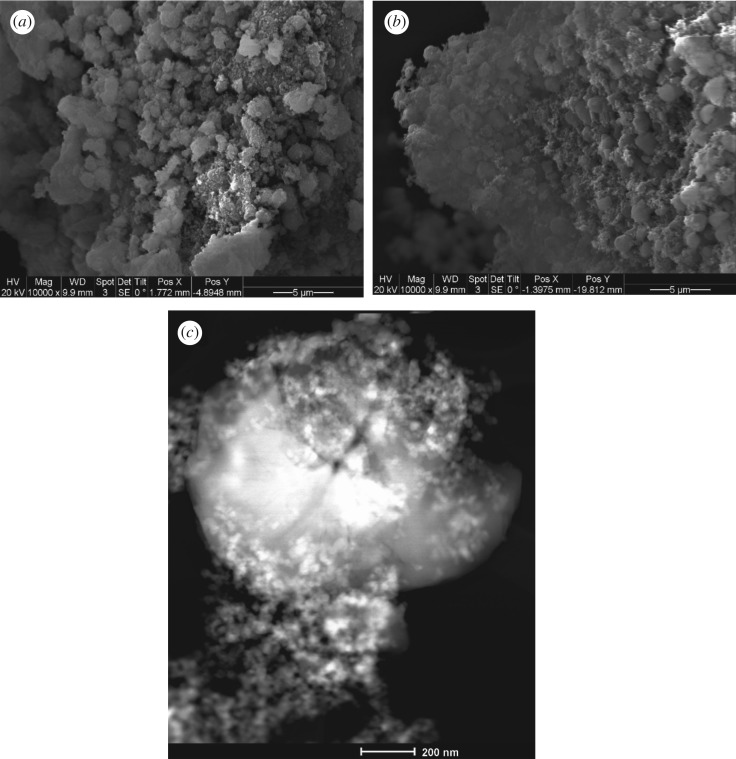
Electron microscope photographs of the composite photocatalysts. (*a*) SEM of PZSB, (*b*) SEM of PZS and (*c*) STEM of PZS.

The composite photocatalyst prepared through binding its components with colloidal silica gel (PZSB) has higher adsorption capacity and lower photocatalytic activity (table 1 and figure 2) than PZS. The suppressed photocatalytic activity might be due to strong adsorption characteristics induced by the colloidal silica binder. Besides, the distribution of the TiO_2_ and ZSM-5 over the silica gel may be a significant factor in this case. Figure 3 illustrates the SEM photographs of the optimum composition of the PZSB and PZS photocatalysts. Figure 3*a,b* indicates that the distribution of the TiO_2_ and ZSM-5 over the silica gel is better for PZS. The better distribution results in higher utilization of the catalyst as pathways for surface diffusion become shorter. However, scanning transmission electron microscope (STEM) of the PZS (figure 3*c*) reveal that developing a uniformly distributed TiO_2_/ZSM-5 supported photocatalyst is still a challenge.

To conclude, although the PZS composite photocatayst has the lowest P25 ratio among the synthesized photocatalysts, it has the highest photocatalytic activity.

### 3.3. Comparing the efficiency of the promising composite photocatalyst to Degussa P25

The photocatalytic efficiency of the PZS was compared to commercial Degussa P25 as a reference point. Table 1 illustrates that the dark adsorption capacity of the PZS composite photocatalyst is twice that of Degussa P25. This means that the chance of contact between phenol and P25 is higher in the PZS. Figure 2*b* and table 1 reveal that the PZS has higher photocatalytic activity than Degussa P25. Also, the percentage of phenol mineralization after 1 h of irradiation using PZS is 1.25 times higher than Degussa P25. This is despite the fact that the TiO_2_ of the composite photocatalyst has hidden surface from the embedding. Adsorb and shuttle appears to have been effective, implying that PZS achieves a precise balance of adsorption capacity, absorption strength and surface mobility.

### 3.4. Photocatalytic ozonation

**Figure 4.**
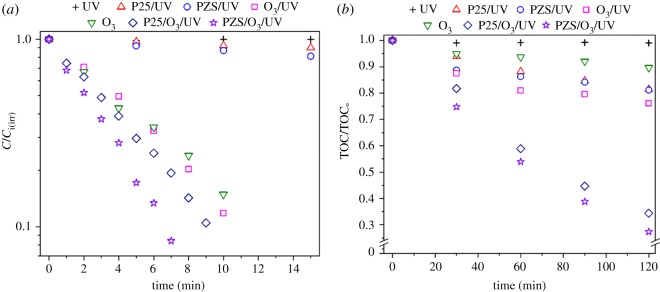
(*a*) Degradation and (*b*) mineralization of SMX with time using PZS and commercial Degussa P25 under different oxidative processes.

The degradation and mineralization of SMX as a function of time under UV, ozone (O_3_), O_3_/UV, Degussa P25/UV, PZS/UV, Degussa P25/O_3_/UV and PZS/O_3_/UV is shown in figure 4*a,b*. The first-order rate constants in addition to the correlation coefficient are tabulated in table 2.

**Table 2.** The first-order rate constant (*k*) and correlation coefficient (*R*^2^) for different oxidative processes.

process	*k* (min^−1^)	*R*^2^
UV	0.001	0.806
P25/UV	0.0074	0.997
PZS/UV	0.0104	0.992
O_3_	0.177	0.990
O_3_/UV	0.1987	0.995
P25/O_3_/UV	0.2369	0.998
PZS/O_3_/UV	0.3675	0.990

Table 2 indicates that the lowest degradation rate was achieved by the photocatalytic degradation process using Degussa P25. This is likely due to a limited supply of SMX from bulk solution to active sites on the P25 surface [27]. However, using PZS as the photocatalyst somewhat overcomes this drawback via the aforementioned ‘adsorb and shuttle’ mechanism and clearly enhances the degradation rate (table 2). On the other hand, a significant increase in the degradation rate was observed by the ozone-based processes (O_3_ and O_3_/UV). Further increase in the degradation rate was observed when ozonation was combined with photocatalysis (P25/O_3_/UV and PZS/O_3_/UV). As far as the degradation of SMX is concerned, O_3_/UV or even O_3_ seems to be better than P25/O_3_/UV (figure 4*a* and table 2) from a practical and economic point of view. But, considering the mineralization of SMX, figure 4*b* shows that P25/O_3_/UV is much better than O_3_/UV.

Table 2 and figure 4*b* show that the highest removal rate and mineralization of SMX were achieved by photocatalytic ozonation using PZS as the photocatalyst (PZS/O_3_/UV). SMX completely disappeared within 10 min. The degradation rate of SMX by photocatalytic ozonation using PZS as the photocatalyst (PZS/O_3_/UV) is about 1.8 and 1.5 times higher than O_3_/UV and P25/O_3_/UV, respectively. Also, SMX mineralization by the PZS/O_3_/UV is 2.9 and 1.1 times higher than O_3_/UV and P25/O_3_/UV, respectively. It has been reported that, in addition to concentrating the organic compounds close to the active site of the TiO_2_, high silica zeolites concentrate ozone in adsorption phase and therefore enhance the degradation and mineralization rate by contacting the dissolved ozone and the organic compound at the zeolite surface [28,29]. This result also shows the merit of the composite photocatalyst (PZS) in the degradation and mineralization of SMX.

## 4. Conclusion

This study aimed at testing the effect of the composite photocatalyst (TiO_2_/ZSM-5/silica gel) supports characteristics and preparation method on its photoactivity. The particle size and pore size of the silica gel and the surface hydrophobicity of ZSM-5 have a significant effect on the performance of the composite photocatalyst. The composite photocatalyst prepared without a binder has higher photoactivity than the one prepared using a binder. In addition, it has better distribution of the catalyst components over the inert support. A photocatalyst that has higher photoactivity and is more easily separable than commercial Degussa P25 was prepared. The photocatalytic ozonation process using easily separable composite photocatalyst PZS accelerates the degradation and mineralization of compounds, copes with the decreased efficiency of immobilized catalysts and makes the process so appealing for practical applications. Finally, more research is needed to develop a preparation method that gives higher uniformity of distribution of TiO_2_/ZSM-5 over an inert support.

## Supplementary Material

Data

## Data accessibility

All data of this paper are available in the electronic supplementary material.
